# Revisiting the Life Cycle of Dung Fungi, Including *Sordaria fimicola*

**DOI:** 10.1371/journal.pone.0147425

**Published:** 2016-02-03

**Authors:** George Newcombe, Jason Campbell, David Griffith, Melissa Baynes, Karen Launchbaugh, Rosemary Pendleton

**Affiliations:** 1 Department of Forest, Rangelands and Fire Sciences, University of Idaho, Moscow, Idaho, United States of America; 2 Environmental Sciences Program, University of Idaho, Moscow, Idaho, United States of America; 3 Palouse Research, Education and Extension Center, University of Idaho, Moscow, Idaho, United States of America; 4 Center for Resilient Communities, University of Idaho, Moscow, Idaho, United States of America; 5 US Forest Service, Rocky Mountain Research Station, Albuquerque, New Mexico, United States of America; Umeå Plant Science Centre, Umeå University, SWEDEN

## Abstract

Dung fungi, such as *Sordaria fimicola*, generally reproduce sexually with ascospores discharged from mammalian dung after passage through herbivores. Their life cycle is thought to be obligate to dung, and thus their ascospores in Quaternary sediments have been interpreted as evidence of past mammalian herbivore activity. Reports of dung fungi as endophytes would seem to challenge the view that they are obligate to dung. However, endophyte status is controversial because surface-sterilization protocols could fail to kill dung fungus ascospores stuck to the plant surface. Thus, we first tested the ability of representative isolates of three common genera of dung fungi to affect plant growth and fecundity given that significant effects on plant fitness could not result from ascospores merely stuck to the plant surface. Isolates of *S*. *fimicola*, *Preussia* sp., and *Sporormiella* sp. reduced growth and fecundity of two of three populations of *Bromus tectorum*, the host from which they had been isolated. In further work with *S*. *fimicola* we showed that inoculations of roots of *B*. *tectorum* led to some colonization of aboveground tissues. The same isolate of *S*. *fimicola* reproduced sexually on inoculated host plant tissues as well as in dung after passage through sheep, thus demonstrating a facultative rather than an obligate life cycle. Finally, plants inoculated with *S*. *fimicola* were not preferred by sheep; preference had been expected if the fungus were obligate to dung. Overall, these findings make us question the assumption that these fungi are obligate to dung.

## Introduction

The life cycle of many coprophilous fungi is thought to be obligate to dung. *Sordaria fimicola* is one such dung fungus that has also long been used to study meiosis [1,2], and to teach genetics [3]. Its products of meiosis, ascospores, form easily in artificial culture. As one of the best known species of the dung fungi, *S*. *fimicola* is thought to have a typical cycle in nature: sexual reproduction obligate to mammalian, and possibly avian [4], herbivore dung after passage through the herbivore’s gastrointestinal (GI) tract [5,6]. Following meiosis on dung, ascospores are discharged and stick onto plant surfaces where they are thought to remain epiphyllous. With this view of dung fungi, plants are little more than a staging ground for reintroduction of spores into an animal’s GI tract [7]. Spores of obligate dung fungi in Quaternary sediments are thus used as proxies, or bio-indicators, of ancient herbivore activity [8], and to some extent, this inference has been verified in present-day, plant communities [4,9,10]. Among the most reliable and most widely used bio-indicators are spores of species of *Sordaria* [7].

Endophyte research has raised questions about life cycles of dung fungi, since many genera [11,12,13,14], including species of *Sordaria* [15], have long been isolated from surface-sterilized plant tissues [16,17,18,19]. However, such isolations do not necessarily imply endophytic presence within plant tissues because ascospores on plant surfaces might resist bleach, alcohol, and other surface-sterilants. In fact, ascospores of some dung fungi do not germinate unless they are exposed to heat [20], chemical cues, or drying [21].

On the other hand, we reasoned that dung fungus inoculants would necessarily have colonized the tissues of plants whose growth and fecundity were affected by that inoculation. The alternative explanation for such effects would be that spores on the plant surface affect plant fitness; we are unaware of a single case reporting such a phenomenon. Significantly, two studies have actually shown that plant fitness can be affected by dung fungi, implying colonization beneath the surface. In one, *S*. *fimicola* reduced dry matter accumulation, root length, and seedling vigor of maize [22]. In the other, positive effects of *S*. *fimicola* on plant growth and defense were observed [23].

After isolating various dung fungi, including *Sordaria fimicola*, from basal nodes of *Bromus tectorum* [14], we performed inoculation experiments. *Bromus tectorum* was inoculated with *S*. *fimicola* and representative isolates of two other genera of dung fungi, *Preussia* and *Sporormiella*, to see if plant growth and fecundity could be affected.

Additional questions motivated the work reported here. We wondered whether grazing animals associated with the spread of *B*. *tectorum* [24,25,26] might also spread dung fungi that could, in turn, regulate populations of this invader. This might be especially true if herbivores preferred plants infected with dung fungi. If dung fungi truly were obligate [7], then this preference might be expected. Given the research prominence of *S*. *fimicola*, we conducted the full suite of investigations with it rather than the *Preussia* or *Sporormiella* isolates. The ability of a single isolate of *S*. *fimicola* to complete its life cycle both in the traditionally envisioned, dung-obligate way and also in host plants, was then tested. Finally, a preference test with a mammalian herbivore (i.e., sheep) was conducted, and a correlate of possible preference (i.e., forage quality) was analyzed.

## Materials and Methods

### Inoculation of *Bromus tectorum* seedlings with representative isolates of three dung fungi

Greenhouse assays were conducted to determine whether *B*. *tectorum* seedlings could be infected by isolates of the following three dung fungi, each isolated from *B*. *tectorum* plants in the field: *Sordaria fimicola* (CID 33 isolate from the Sandia Mountains in New Mexico, GenBank accession HQ829068), *Preussia* sp. (CID 34 isolate from eastern Washington), and *Sporormiella* sp. (CID 329 isolate from the Sandia Mountains, GenBank HQ829154) [14]. Each isolate was identified on the basis of morphology. The ITS sequence of CID 33 was 100% identical to FN868475, a GenBank accession corresponding to an isolate of *S*. *fimicola* from *Pinus halepensis* reported as endophytic [27]. The three isolates were used to inoculate seedlings from three populations of *B*. *tectorum* in which seed had been collected: 1) Zia Indian Reservation, New Mexico, 2) McCroskey State Park, Idaho, and 3) Kendrick, Idaho. Vernalized seeds were surface-sterilized in 50% ethanol for five minutes, rinsed for one minute in sterile distilled water (SDW) [15,28] and placed onto sterile blotter paper in Petri dishes for germination. Germinants (seedlings) were inoculated by placing them in direct contact with mycelium of PDA cultures of each of the three inoculants. Contact was maintained for 24 hours. Controls were in contact with sterile PDA for 24 hours. Three seedlings were planted in each 10 cm^2^ pot pre-filled with sterile potting mix (Sunshine Mix #1, Sun Gro Horticulture Inc., Bellevue, WA, USA). Thinning at two weeks left one seedling per pot. Each combination of treatment (4, including control) and plant population (3) was replicated with 20 seedlings (total of 240 seedlings), and the experiment was conducted as a randomized complete block design. Plants were grown in the greenhouse (18-hour days at 25°C, 6-hour nights at 20°C) for four months at which point aboveground biomass was harvested, separated into seed and vegetative lots, dried for 72 hours at 60°C, and weighed. Fecundity and growth were measured as the number of seeds and vegetative biomass, respectively. Data were analyzed with a GLM model and Bonferroni pairwise comparisons within SysStat 12.02.00.

### Inoculation of roots or leaves of *B*. *tectorum* seedlings with *S*. *fimicola*

#### Root inoculations

Fifty seedlings of *B*. *tectorum* were placed into new Petri dishes containing *S*. *fimicola* ‘CID33’ such that there was direct root-mycelium contact for 48 hours; 50 control seedlings were placed onto sterile PDA for the same period of time. Following inoculation, seedlings were removed from each treatment and placed on moistened, sterile filter paper in sterile Petri dishes. After one week, seedlings were removed from the Petri dishes, divided into leaf and root tissue and surface-sterilized. Tissue segments were placed onto sterile PDA in separate Petri dishes to facilitate the re-isolation of *S*. *fimicola* ‘CID33’. Observations were made daily; all emerging endophytes were recorded, isolated and cultured. The assay was conducted under ambient laboratory conditions (20°C with 14-hour days).

#### Leaf inoculations

Fifty seedlings were planted in each of two 26x52 cm trays filled with sterile Sunshine Mix #2 potting soil, and placed in the greenhouse. Inoculum was a suspension of mycelium and fruiting bodies of *S*. *fimicola* ‘CID33’ from two Petri plates (8.5 cm diameter) in SDW. Both cultures were scraped into 100 mL of SDW and vigorously shaken to disperse the fungal material throughout the solution [29]. Inoculant was transferred to a hand-held spray bottle. A second spray bottle was filled with 100 mL of SDW. Seedlings were approximately five cm tall when first inoculated. One tray of fifty seedlings was inoculated with the suspension and the control was mock-inoculated with SDW. A second inoculation was conducted 48 hours later; 50 mL of inoculant was used for each inoculation assay. After each inoculation, trays were bagged for 18 hours to facilitate infection of moist plants. Plants remained in the greenhouse for one week following the second inoculation. Plants were then harvested, divided into leaf and root tissue and surface-sterilized. Sterilized leaf and root tissue were placed on sterile PDA in separate Petri plates to facilitate fungal growth. Observations were made daily; all emerging endophytes were recorded, isolated and cultured.

### Effects of *S*. *fimicola* on forage quality attributes

Forage quality was assessed with 100-g samples from ten plants of each treatment, taken daily for eight days, while plants were flowering and starting to produce seeds. Within 30 minutes following collection, samples were heated to 70°C in a microwave oven, to curtail metabolic activity [30]. Samples were then dried at 50°C in a forced-air oven for 48 hours, and submitted to Dairy One Laboratories, Ithaca, NY, for analysis. Analyses included crude protein (CP), and acid and neutral detergent fiber content (ADF and NDF). CP was assessed using a Leco FP-528 Nitrogen/Protein analyzer. For ADF and NDF, samples were digested, in a solution appropriate to each fiber type, in an ANKOM A200 Digestion Unit.

### Completion of life cycle via passage through an herbivore and reproduction in dung

In late winter, four open ewes of the Suffolk breed were placed in a 2 x 3 m indoor pen, with *ad libitum* access to water. For two days, all sheep were fed a daily ration of 2.25 kg alfalfa pellets per sheep. Alfalfa pellets were utilized as a purgative to remove pre-existing fungi, including *S*. *fimicola* [31,32,33,34], in the GI tracts of sheep. All feed was heated, in a conventional oven, to 50°C for 30 minutes. Feed was also heated to kill viable fungal material inherent in the feed. On the third day, sheep were switched to a daily diet of 2.25 kg beet pulp pellets, heated to 50° for 30 minutes before feeding. The CID 323 isolate of *S*. *fimicola* was incubated, on PDA, in four standard 100-mm petri dishes for 21 days at 20°C. Spores and mycelia from these plates were combined, homogenized, and suspended in SDW at 20°C. In the morning of the 7^th^ and 8^th^ days of the experiment all sheep were dosed, via esophogeal tube, with 200 ml of this solution.

Prior to *S*. *fimicola* dosing, fecal samples were collected from each ewe, via rectal palpation, once daily. Following dosing, collection of samples occurred twice per day, until the end of the experiment on the 11^th^ day. All fecal samples were incubated and assessed for presence of *S*. *fimicola* fruiting bodies.

### Completion of life cycle via infection of a plant and reproduction in senescent plant tissue

Ten *B*. *tectorum* seedlings were inoculated with the CID 323 isolate of *S*. *fimicola* ten days after emergence. Inoculum was produced by covering two, 21-day-old cultures on PDA with SDW, agitating with a sterile glass rod, and diluting the resulting spore and mycelial suspension to 100 mL. Inoculum was applied to the *B*. *tectorum* seedlings with a hand-held spray bottle, and seedlings were then bagged overnight (18 hours) to facilitate infection. Seedlings were grown in the greenhouse for 10 days (14:10 photoperiod with 25°C day and 20°C night mean temperatures) after the inoculation event.

The aboveground tissue of five plants was harvested from the greenhouse and surface- sterilized (according to protocol described above) in the laboratory. Five leaf sections from each plant were plated on PDA to confirm infection, and impression plates were used to confirm surface-sterilization. Remaining leaf tissue was placed on autoclaved, washed sand in sterile plastic bags. Five mL of SDW was added to each bag, and the resulting growth chambers were sealed. Growth chambers were incubated at laboratory conditions (20°C and 10:14 photoperiod) for 21 days, with daily observations.

### Reproduction in the absence of competitors in plant tissue

Leaf tissue from five *B*. *tectorum* plants was collected after 60 days of growth (14:10 photoperiod with 25°C day and 20°C night mean temperatures). Leaf tissue (10–20 cm long leaf sections attached to stems) was autoclaved on a Liquid-20 cycle (20 min sterilization and slow exhaust). Sterilized *B*. *tectorum* leaf tissue was dipped into a spore and mycelial suspension of *S*. *fimicola* (CID323) for 5 seconds, placed on sterile paper towels, moistened with 5 mL of SDW, and placed in sterile plastic bags. Bags were sealed and incubated at ambient laboratory conditions (20°C and 10:14 photoperiod), with daily observations for 21 days.

### Preference experiment

Six yearling Suffolk ewes (provided by the University of Idaho Palouse Research, Education, and Extension Center) were housed in a communal pen with *ad libitum* access to water and locally grown grass hay, comprised primarily of tall fescue (*Festuca arundinacea*). Hay was removed each evening. The preference trial lasted 12 days and consisted of two phases: a protocol conditioning phase during the first four days, followed by preference testing. The protocol conditioning phase familiarized the animals with the preference trial procedure using daily treatments over a four-day period. Each morning sheep were placed in individual pens, and each sheep was offered 100 g of each of smooth brome (*Bromus inermis*), and meadow foxtail (*Alopecuris pratensis*), both of which were familiar to the sheep subjects. All feed samples were gathered from nearby pastures and material was hand-chopped into segments less than four cm in length. All feeds were collected each morning, and offered in a fresh state. Feeds were offered in 14x14x16 cm plastic containers placed adjacent to one another. Containers were removed when 90% of one feed, by visual estimate, had been consumed from one container, or when one hour had passed. Preference was indicated by consumption of one grass at a rate greater than 60% of the total amount of both grasses offered. Avoidance was ascribed to samples that constituted consumption of 40% or less of the total amount of grass offered.

The eight days of daily preference began on the 23^rd^ of June, 2011. The presentation protocol was identical to that of the pre-conditioning phase. *B*. *tectorum* samples were collected from *S*. *fimicola* CID323-infected and control plants established in the field. For the first five days, each sheep received 100 g of each of infected (S^+^) and uninfected (S^-^) *B*. *tectorum*. On the sixth and seventh days sheep received 150 g of each and, on the final day, 200 g of each. Preference or avoidance was measured as the daily intake of S^+^ *B*. *tectorum*, expressed as a percentage of total daily consumption of *B*. *tectorum* (S^+^ + S^-^). Preference was indicated by consumption of one grass at a rate greater than 60% of the total amount of both grasses offered. Avoidance was ascribed to samples that constituted consumption of 40% or less of the total amount of grass offered.

The percent consumption of S^+^ *B*. *tectorum* for all ewes was evaluated in a repeated measures analysis of variance with daily tests as the repeated variable (Prism Version 5.04 software, GraphPad 2010). An arcsine square root transformation was performed to normalize the percentage data [35].

### Ethics Statement

Experiments with sheep were carried out in strict accordance with the recommendations in the Guide for the Care and Use of Laboratory Animals of the National Institutes of Health. The protocol was approved by the Institutional Animal Care and Use Committee of the University of Idaho (protocol #2011–7).

## Results

### Inoculation of *Bromus tectorum* seedlings with representative isolates of three dung fungi

**Fig 1:**
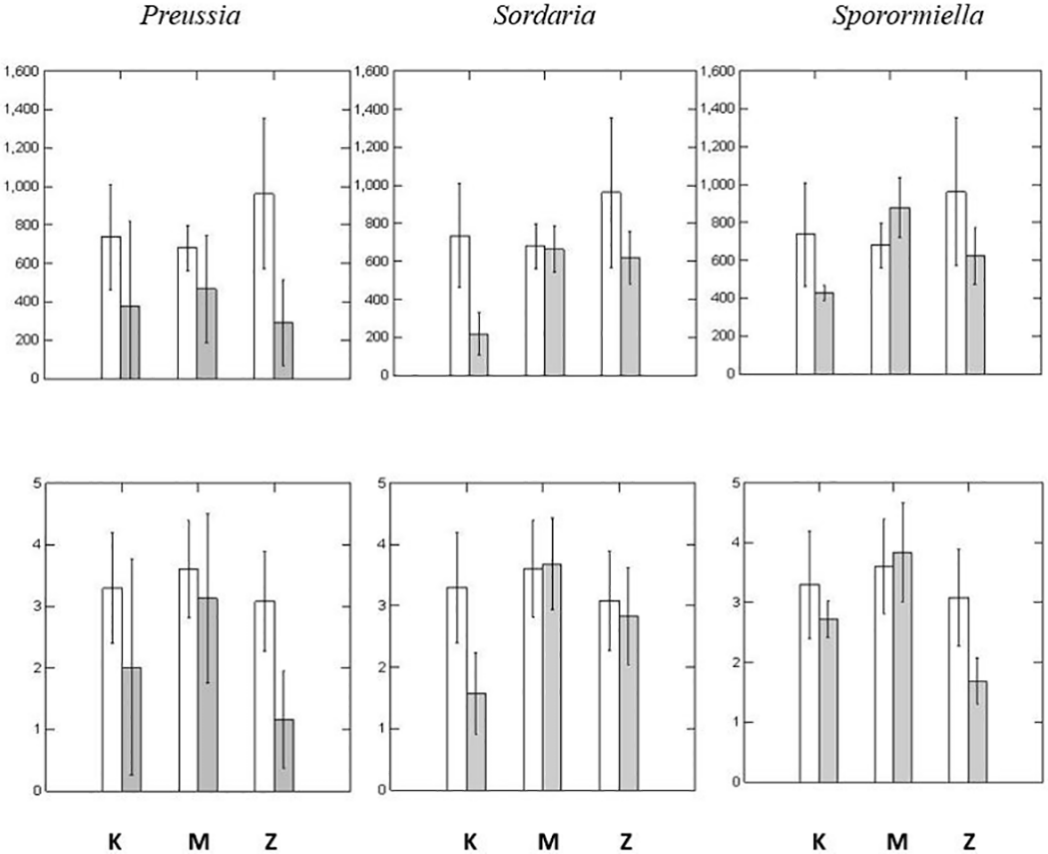
Fecundity (top row—seeds per plant) and growth (bottom row—grams of dry, aboveground, vegetative biomass per plant) of some populations of *Bromus tectorum* were significantly reduced by inoculation with representative isolates of three genera of dung fungi (i.e., *Preussia*, *Sordaria*, and *Sporormiella*). Clear bars: uninoculated controls. Shaded bars: inoculated. K, M, and Z were populations of *B*. *tectorum* from seed collected in Kendrick, Idaho, McCroskey, Idaho and Zia, New Mexico, respectively. Fecundity and growth of Zia plants were negatively affected by *Preussia* CID 34 (P = 0.001 and P = 0.009, respectively) and *Sporormiella* CID 329 (P = 0.006 and P = 0.029, respectively); fecundity and growth of Kendrick plants were negatively affected by *Sordaria fimicola* CID 33 (P = 0.001 and P = 0.001, respectively).

Inoculations resulted either in significant reductions in growth and fecundity or in insignificant effects; there were no significant increases in growth and fecundity as a result of inoculations with the three dung fungi (Fig 1). Each of the three fungi reduced growth and fecundity of one of the three plant populations but none had any effect on plants from the McCroskey State Park population. Both fecundity and growth of the Kendrick plants were negatively affected by *S*. *fimicola* CID 33. Similarly, Zia plants were negatively affected by the other two isolates of dung fungi (*Preussia* CID 34, and *Sporormiella* CID 329).

### Inoculation of roots or leaves of *B*. *tectorum* seedlings with *S*. *fimicola*

#### Root inoculations

*Sordaria fimicola* CID 33 was observed in 80% and 50% of root and leaf tissues, respectively, that were subjected to the re-isolation protocol. This fungal inoculant was only found on leaf pieces from seedlings whose roots also yielded *S*. *fimicola*. Controls were free of *S*. *fimicola*.

#### Leaf inoculations

As with root inoculations, *S*. *fimicola* CID 33 was observed in most samples of root and leaf tissues in which re-isolation was attempted: 86% and 76%, respectively. Again, this fungal inoculant was only found on root pieces from seedlings whose leaves also yielded *S*. *fimicola*. Controls were free of *S*. *fimicola*.

### Effects of *S*. *fimicola* on forage quality attributes

Crude protein and fiber were not affected by infection with the CID 323 isolate of *S*. *fimicola*. Crude protein in both S+ and S- plants averaged 11.5% with a standard error of 0.39, while the mean acid and neutral detergent fiber contents were 34.5% and 55%, with standard errors of 0.58 and 0.89, respectively (Table 1 and S1 Table).

**Table 1:** Mean comparisons of forage attributes of *Sordaria*-infected (S+) and uninfected (S-) *B*. *tectorum*.

	Crude Protein		Acid Detergent Fiber		Neutral Detergent Fiber		Total Digestible Nutrients	
	S+	S-	S+	S-	S+	S-	S+	S-
**Mean**	12.00%	10.94%	34.44%	34.53%	54.63%	56.13%	60.00%	59.63%
**Standard Error**	0.46	0.61	0.67	1.00	1.24	1.32	0.38	0.32
**T Statistic**	1.39		-0.07		-0.83		0.75	
**P Value**	0.19		0.94		0.42		0.46	

### Completion of life cycle via passage through a herbivore and reproduction in dung

**Fig 2:**
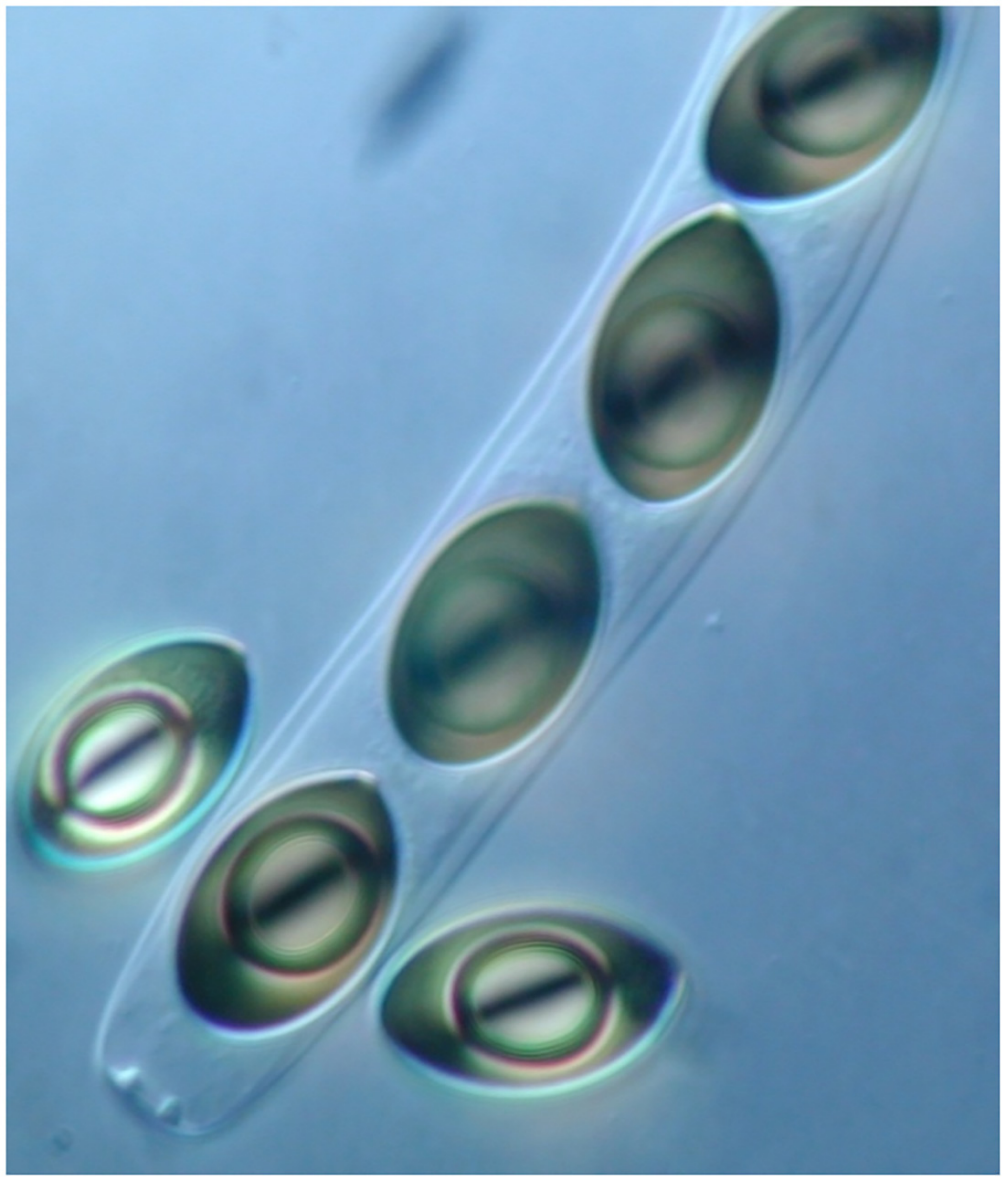
The sexual state observed both after passage through a sheep, and after infection of a *B*. *tectorum* plant. Ascospores, the products of meiosis, within and outside an ascus of *S*. *fimicola* CID 323.

All sheep dosed with the *S*. *fimicola* CID 323 suspension via esophogeal tube produced dung which was positive for perithecia, asci and ascospores of *S*. *fimicola* (Fig 2) between 24 and 72 hours following initial dosing on the seventh day (Table 2). All four subjects produced dung positive for *S*. *fimicola* at some point following dosing. Following the feeding of alfalfa pellets, and prior to dosing, no subjects produced dung positive for *S*. *fimicola*.

**Table 2:** Transit of *S*. *fimicola* CID 323 through the gastrointestinal tracts of sheep after dosing with live cells of the fungus via esophogeal tube.

Hours (from initial dose)	Sheep Subject				Dosing
-168	**A**	**B**	**C**	**D**	
-144	**-**	**-**	**-**	**-**	
-120	**-**	**-**	**-**	**-**	
-96	**-**	**-**	**-**	**-**	
-72	**-**	**-**	**-**	**-**	
-48	**-**	**-**	**-**	**-**	
-24	**-**	**-**	**-**	**-**	
0	**-**	**-**	**-**	**-**	Dosing
24	**-**	**-**	**+**	**-**	
36	**+**	**-**	**-**	**-**	
48	**na**	**-**	**na**	**-**	
60	**-**	**-**	**+**	**+**	
72	**-**	**+**	**+**	**-**	
84	**-**	**-**	**+**	**-**	
96	**na**	**-**	**+**	**-**	
108	**na**	**-**	**na**	**-**	

**+**, *S*. *fimicola* fruiting in dung; **-**, no fruiting; **na**, no sample collected.

### Completion of life cycle via infection of a plant and reproduction in senescent plant tissue

Two of the five *B*. *tectorum* plants that were inoculated with the CID 323 isolate of *S*. *fimicola* were infected as suggested by re-isolation. Perithecia began developing on the leaf tissue of one plant after 17 days. These began to eject spores after 21 days. Identity as *S*. *fimicola* was confirmed by microscopic examination of perithecia, asci and ascospores (Fig 2).

### Reproduction in the absence of competitors in plant tissue

**Fig 3:**
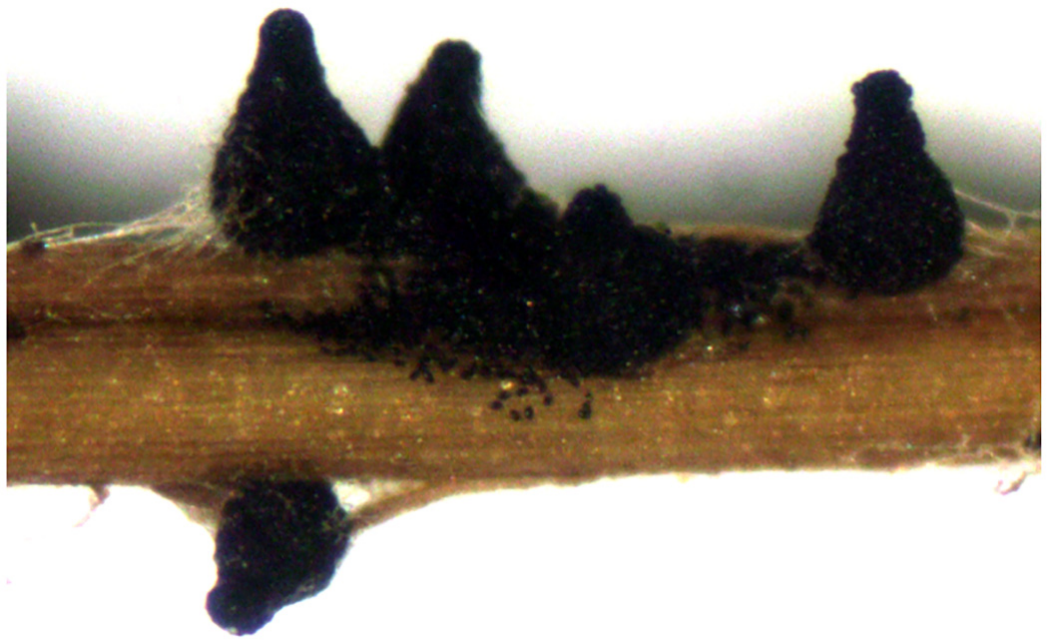
Abundant reproduction on plant tissues. Mature perithecia of *S*. *fimicola* CID 323 on autoclaved, senescent *B*. *tectorum* leaf tissue.

Mycelia developed on sterilized *B*. *tectorum* leaf tissue incubated in sterile conditions within 7 days of inoculation, and perithecia developed after 10 days. Mature *S*. *fimicola* perithecia began ejecting spores by 13 days after inoculation, at which time perithecia were abundant (Fig 3).

### Preference experiment

**Fig 4:**
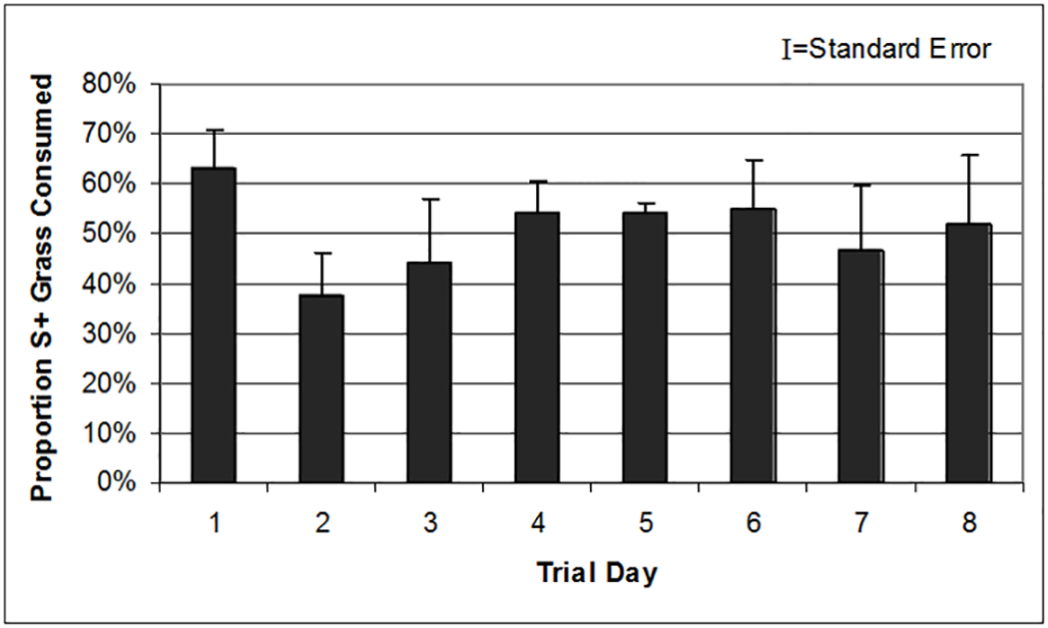
No preference for plants infected with *S*. *fimicola* CID 323 was exhibited by sheep. Mean daily consumption of *Sordaria*-inoculated (S+) *B*. *tectorum* expressed as the percentage of total consumed when six sheep were presented with equal amounts of S+ and S- (uninoculated, control) material.

During the conditioning phase, sheep expressed a significant preference for smooth brome over meadow foxtail (P = 0.0094). Given a hypothesized mean of 50% brome consumption, the sheep exceeded this by 29% and 13% on the third and fourth days, respectively. During the preference trial phase, sheep readily consumed both *Sordaria*-infected (S+) and control (S-) *B*. *tectorum* (S1 Table). In other words, sheep neither preferred nor avoided S+ *B*. *tectorum* (P = 0.7767; Fig 4).

## Discussion

Dung fungi are associated with diverse plant taxa. For example, isolations of *S*. *fimicola* from tissues of at least 24 diverse plant taxa are known [36]. *Preussia* and *Sporormiella* are also commonly reported as endophytes [14,17,37,38,39,40,41]. Past reports of dung fungi as endophytes would seem to challenge the view that their ascospores can be used as proxies for Quaternary herbivore activity. However, those reports have been questioned because surface-sterilization protocols could have failed to kill dung fungus ascospores stuck to the plant surface. Isolation into medium after surface-sterilization would have then misleadingly suggested to researchers that dung fungi were endophytes.

Our focus here was thus on effects of inoculation with dung fungi on plant growth and fecundity. We reasoned that significant effects on plant fitness could not result from ascospores merely stuck to the plant surface. Now, having found that isolates of *S*. *fimicola*, *Preussia* sp., and *Sporormiella* sp. can significantly reduce growth and fecundity of *Bromus tectorum*, we infer that at least these dung fungi can be endophytic and that they are not obligate to dung.

We hypothesized that fungi obligate to dung might be expected to influence plant growth in such a way as to increase palatability to herbivores. We now see that this hypothesis is without merit because the dung fungi that we tested were not obligate to dung.

Spores of dung fungi in Quaternary sediments should not be taken as indisputable evidence of past herbivory. Dung fungus ascospores may be more common in dung than on plant residues in nature, but that difference is unconfirmed. If equally true in the past, that difference might be the basis for continued use of dung fungus spores as bio-indicators of past herbivory. But that use would not be because these fungi have a life cycle obligate to dung.

## Supporting Information

S1 Table Data from forage quality and preference experiments. (XLS)
